# Nickel(II) *N*-Heterocyclic Carbene Complex for the Hydrogenation of 2-Acetylpyridine under Mild Conditions

**DOI:** 10.3390/inorganics11030120

**Published:** 2023-03-14

**Authors:** Mitu Sharma, Amanda M. Perkins, Alison K. Duckworth, Emily J. Rouse, Bruno Donnadieu, Bhupendra Adhikari, Sean L. Stokes, Joseph P. Emerson

**Affiliations:** Department of Chemistry, Mississippi State University, Starkville, MS 39762-9573, USA

**Keywords:** *N*-heterocyclic carbene (NHC), nickel(II)-NHC complex, hydrogenation, 2-acetylpyridine

## Abstract

Catalyst-mediated hydrogenation of ketones via hydride transfer can be directly used in the synthesis of alcohols which can exhibit great potential in the practical synthesis of pharmaceuticals. The application of Ni-NHC complexes in the hydrogenation of ketones is still limited. In a pursuit to study the effect of Ni-NHC-based complexes in the reactivity towards hydrogenation, we have studied the catalytic efficiency of a pendent-type nickel complex [Ni(NHC)_2_](PF_6_)_2_ constructed from a benzimidazole moiety. The hydrogenation of 2-acetylpyridine was studied with respect to catalyst loading, reaction temperature, reaction time, and solvent medium. The complex was broadly characterized by X-ray crystallography, ESI-MS, NMR, UV-Vis, and IR spectral studies.

## 1. Introduction

Nickel is a relatively cheap, and minimally toxic, transition metal, which is an attractive substitute for more expensive transition metals traditionally used in catalysis [1,2]. Nickel has several accessible oxidation states, and is generally more nucleophilic than other d^8^ metal ions [3,4]. This affords an opportunity to activate C─C, C─O, and C─H bonds for oxidative processes. Coupling nickel ions with strongly donating ligands can improve both the reactivity and stability of these reactions [5]. *N*-heterocyclic carbenes (NHCs) are attractive ligand systems to support homogeneous catalysis [6]. After the initial synthesis of the NHCs by Arduengo [7], Lappert and Sellmann demonstated the synthesis of Ni-NHC complexes from electron-rich alkenes and functionalized saturated NHCs [8,9]. These reports are key stepping stones towards developing the scope of the synthetic methods to prepare nickel *N*-heterocyclic carbene complexes, and subsequently their applications in homogeneous catalysis. The scope of reactions that Ni─NHC complexes can catalyze depends on their stability and reactivity, where the bonding character between the carbene carbon and the nickel ion has a direct impact on the electronics of the complex [10]. A previously reported computational study suggest that the Ni─NHC interaction is primarily σ-bonding in nature where electron donation takes place from the NHC ligand to the nickel center [11]. Furthermore, there are important developments in homogeneous catalysis based on employing bulky NHC ligands and nickel *bis*-NHC complexes of the general formula [Ni(NHC)_2_]X_2_ (X = anion) as catalysts [5,10].

The formation of alcohols by hydrogenation of their corresponding carbonyl compounds has become an important class of organic reactions used in modern synthetic chemistry [12,13]. Among the various methods applied for hydrogenation, catalytic transfer hydrogenation reactions find immense applications in chemical industries for the manufacture of fine chemicals, pharmaceuticals, perfumes, and agrochemicals [14,15]. The reduction of the ketones to form alcohols mainly involves the use of the stoichiometric amount of hydride reagents such as H_2_, NaBH_4_, LiAlH_4_ or via transfer hydrogenation using metal-based coordination complexes of platinum, ruthenium, iridium, and rhodium [15]. These reducing agents are restricted to industrial applications owing to numerous disadvantages, for instance, high cost, toxicity, being hazardous, volatility, high reactivity, air and moisture sensitivity, and non-viability for large-scale synthesis [16]. Furthermore, for practical industrial applications, the catalyst used should possess high efficiency, and stability under wide reaction conditions and should follow direct synthetic routes [16].

Specifically, the direct hydrogenation of 2-pyridinyl ketones remains a longstanding challenge due to the coordination effect of pyridine. In 2001, Noyori et al. reported the hydrogenation of 2-acetylpyridine with *trans*-RuCl_2_[(R)-xylBINAP]-[(R)-daipen] [17]. In 2015, Zang et al. reported the use of [Rh(COD)binapine]BF_4_ catalyst for the symmetric hydrogenation of 2-pyridinyl ketones [18]. However, the use of earth-abundant, transition metal catalysts for the hydrogenation of pyridinyl ketones has not been well studied. Here, we report the application of a nickel(II)-NHC-based complex for the catalytic hydrogenation of 2-acetylpyridine to its corresponding alcohol under mild conditions using phenylsilane as the reducing agent. The complex shows good stability at room temperature and under aerobic conditions. This catalytic hydrogenation method can be directly used in the synthesis of alcohols in moderate yields, exhibiting a potential role as a modern and sustainable organic method.

## 2. Results and Discussions

**Scheme 1.**
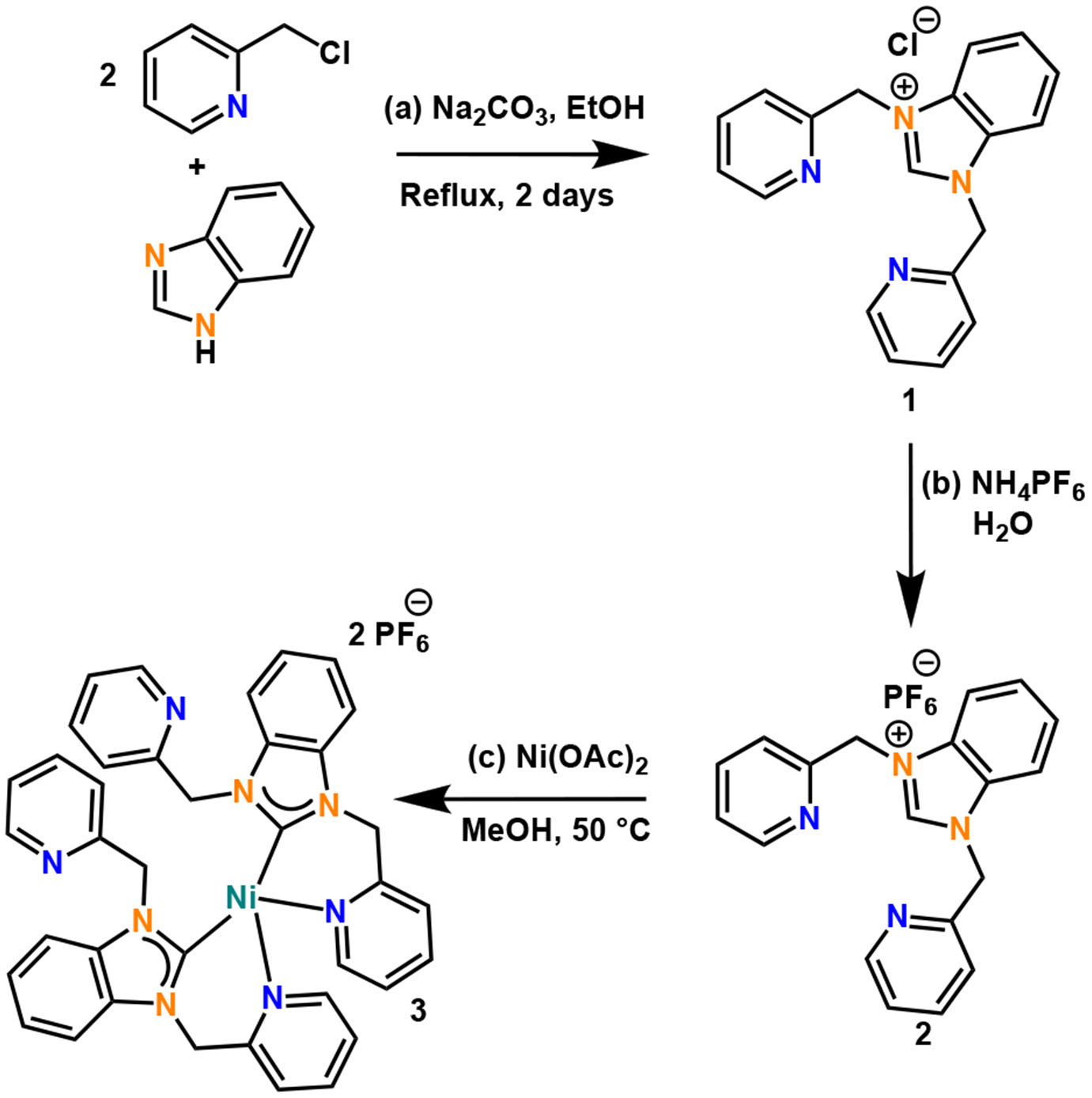
Reaction scheme for (a) ligand generation, (b) ion metathesis to PF_6_^−^ salt, and (c) carbene generation and metal ion ligation in the formation of the nickel(II)-NHC complex (**3**).

The nickel(II)-NHC complex (**3**) was prepared in a two-step procedure by synthesizing the tridentate NHC ligand, as shown in Scheme 1, following the procedure reported by our group previously (SI) [19].

The two-step method involves the reaction of 2 equivalents of picolyl chloride with 1 equivalent of benzimidazole and heating the mixture to reflux in ethanol for 48 h. The tridentate ligand generated (**1**) was in good yields (83%) and the chloride anion was replaced with PF_6_^−^ to form the compound **2**. Compound **2** was dissolved in methanol and a solution of Ni(OAc)_2_·4H_2_O in methanol was added to it dropwise under constant stirring at 50 °C for 1 h. A clear brown solution was obtained, and brownish-yellow crystals of complex **3** were isolated from this solution in good yields (61%). The crystals of complex **3** were further characterized by X-ray crystallography, ESI-MS, and other spectroscopic techniques including UV-Vis, ^1^H NMR, ^13^C NMR, and FT-IR.

**Figure 1.**
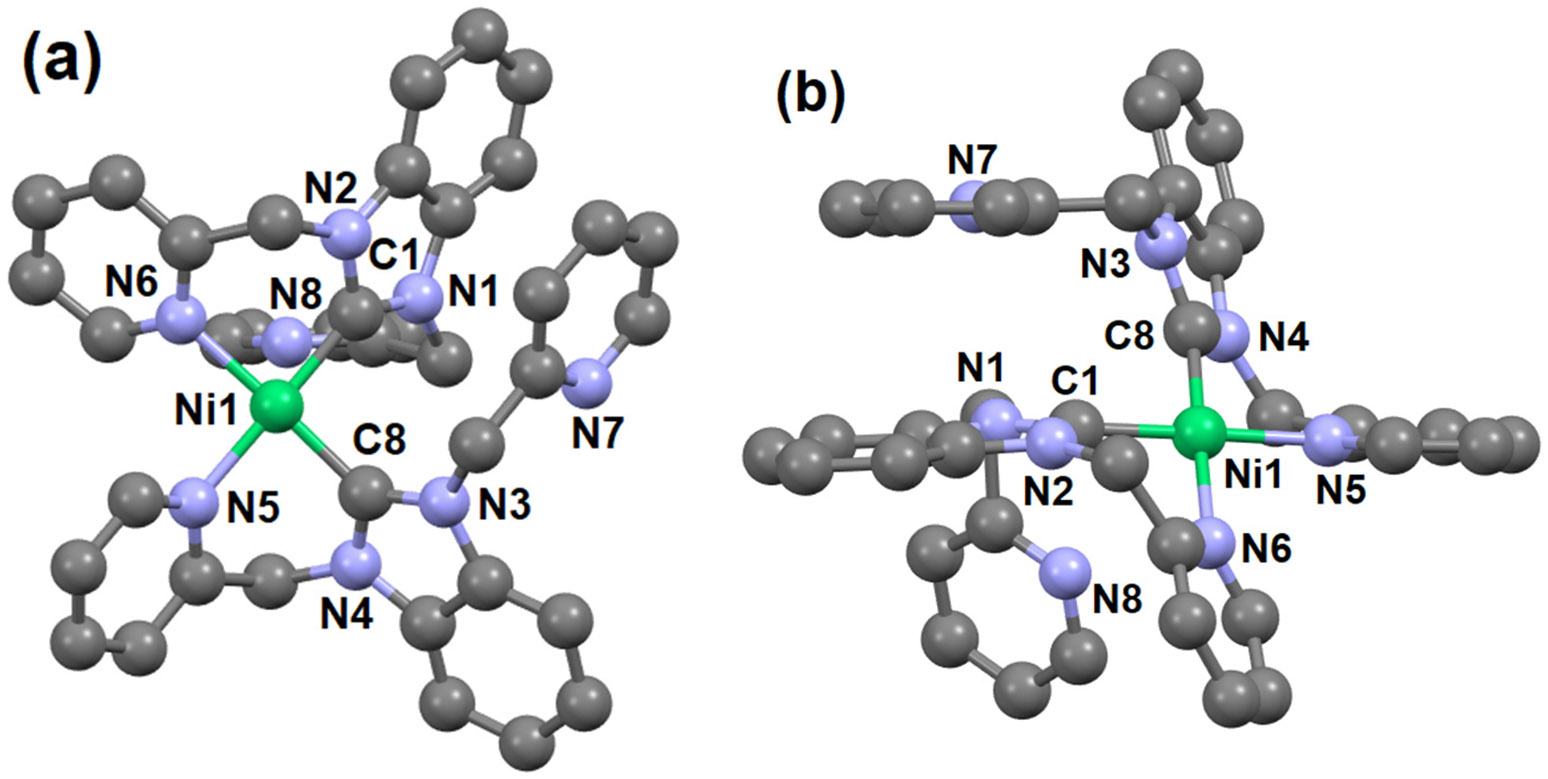
(**a**) and (**b**) ball and stick representation of the crystal structures **3** in different orientations. Hydrogen atoms, methanol, and hexafluorophosphates are omitted for clarity.

Single crystals of **3** suitable for X-ray diffraction were isolated using slow evaporation techniques. The ball and stick representation of **3** is shown in Figure 1 (Figures S1 and S2). The crystallographic and refinement data are shown in Tables S1-S8, and the selected bond lengths and bond angles are listed in Table 1.

The crystallographic asymmetric unit of **3** contains the complex, two hexafluorophosphate anions, and one methanol. The Ni(II) center is four-coordinate by two nitrogen atoms and two carbon atoms from two different NHC ligands in a square planar geometry. The two NHC ligands are bound to the nickel center in a bidentate C, N fashion with C and N donor atoms in *cis* pesitions. The Ni—C dislances 1.871(7) Å and 1.879(7) Å for Ni1—C1 and Ni1—C8 in **3** are consistent to other reported tetra-coordinated square planar complexes, which have an average bond distance of 1.86 Å [5,10,20]. The octahedral complex of 1-((1-benzyl-1H-1,2,3-triazol-4-yl)methyl)-3-(pyrimidin-2-yl)-2,3-dihydro-1H-benzo[d]imidazole, with Ni-C bond distances of 1.991(4) and 1.996(4) Å, is significantly longer than some of the known square-planar Ni─NHC complexes [20,21]. The square planar geometry of the **3** was evaluated based on the equation reported by Yang et al. [21,22]. The two largest angles [C8-Ni1-N6 = 177.9(3) and N5-Ni1-C1 = 179.1(3)] (Table 1), resulting in a τ4 value of 0.02, indicates that the geometry of **3** is very close to ideal square planar with negligible distortions.

**Figure 2.**
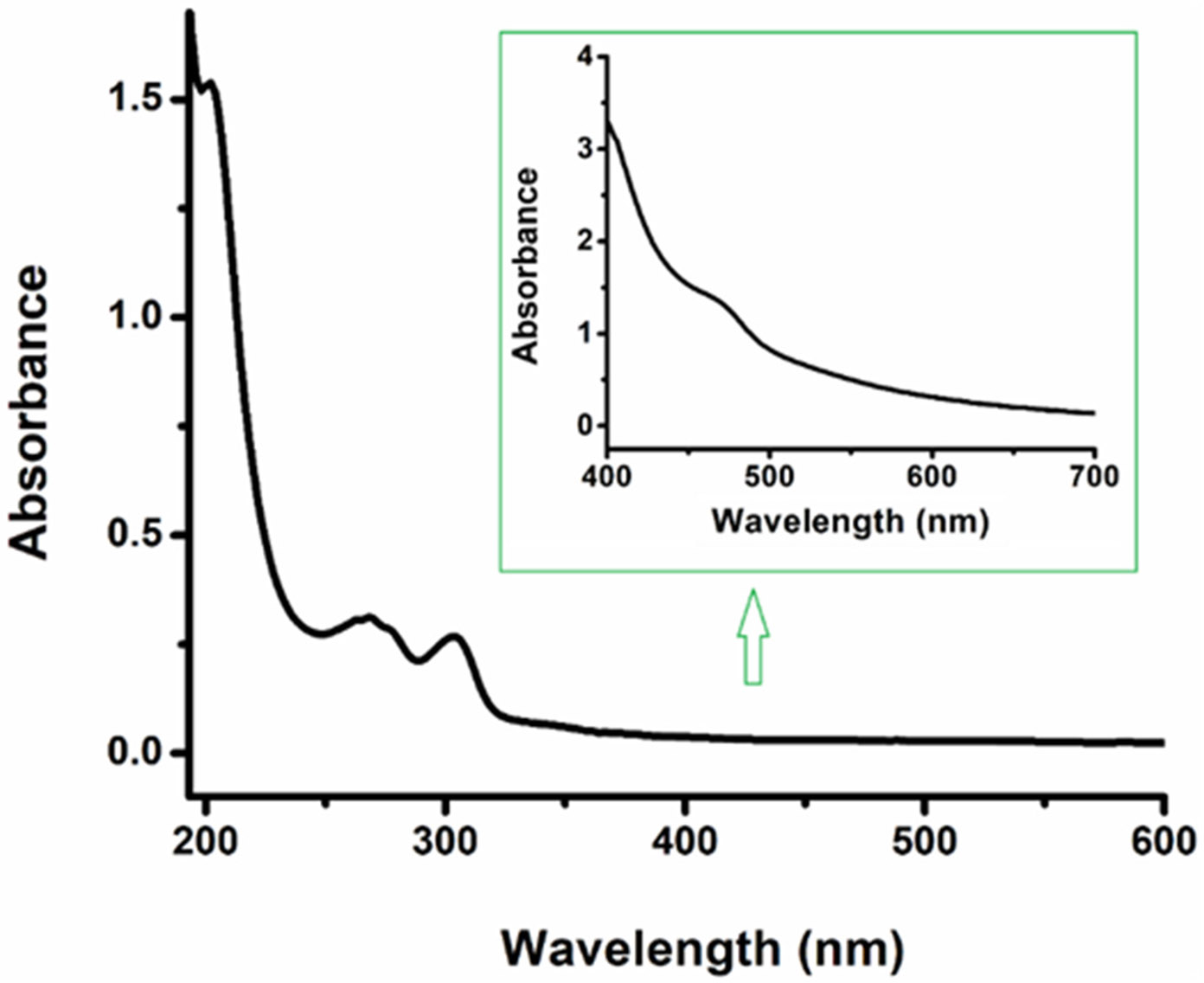
UV–Vis absorption spectra of complex **3** measured in CH_3_CN (conc: 5 × 10^−6^ M; Inset conc: 5 × 10^−3^ M).

The UV-Vis for complex **3** was measured in acetonitrile. A d–d transition band (λ_max_ = 470 nm; ε_470_ 273 M^−1^cm^−1^) was observed, which is consistent with the splitting of d orbitals due to the strong field nature of the NHC ligands stabilizing the t_2g_ orbitals in the complex (Figure 2). The absorption bands attributed to the ligand-based π—π* transition in these complexes were observed at λ_max_ = 202 nm, ε_202_ = 192,500 M^−1^cm^−1^ and 266 nm, ε_266_ = 38,500 M^−1^cm^−1^. As shown in Figure 2, a strong band centered at about λ_max_ = 303 nm and ε_303_ = 33,125 M^−1^cm^−1^ was observed. This band could arise from either a ligand-to-metal charge transfer (LMCT) or a π−π* transition, but further interrogation is required to make this assignment.

The quantitative IR spectroscopic data for ligand **2** and complex **3** were recorded without specific isotopic assignments (Figure S3). For ligand **2,** the vibrational modes consistent with C═N bonds were observed at 1594 cm^−1^, which is similar to some previously reported N-heterocyclic carbenes. A visible shift in the C═N vibrational region was observed at 1609 cm^−1^ upon the coordination of nickel(II) to the NHC ligand in complex **3**. This result is consistent with other reported NHC complexes [23].

Hydrogenation of 2-acetylpyridine using phenylsilane as the reducing agent was achieved in methanol catalyzed by the newly synthesized complex **3** in catalytic amounts. The effect of various parameters such as catalyst concentration, solvent type, temperature, and time were evaluated to optimize the reaction conditions for maximum transformation of 2-acetylpyridine.

In an initial reaction, 2 mol % of catalyst **3** was used for preliminary screening of its efficiency in the hydrogenation of 2-acetylpyridine, in the presence of phenylsilane as the reducing agent under N_2_ condition for 24 h at room temperature. It was observed that 31% of observable amounts of 1-(pyridin-2-yl)ethan-1-ol were formed after 24 h of reaction time under room temperature and inert conditions in methanol (Table 2, entry 1). To optimize the reaction conditions for the maximum hydrogenation of the ketone, the effect of the catalyst amount was first evaluated under identical conditions. Under these conditions, it was observed that when the catalyst loading was increased from 2 to 10 mol %, there was a gradual increase in the % conversion from 31% to 40% until a catalyst loading of 6 mol % was reached (Table 2, entries 2 and 3), as evident from the GC measurements (Figure S4). This could be due to the increase in available reaction centers in the reaction mixture as the catalyst loading is increased gradually. Further increase in catalyst loading to 8 and 10 mol % did not show any enhancement in the % conversion of 1-(pyridin-2-yl)ethan-1-ol which may have occurred due to the saturation of active metal centers in the reaction medium (Table 2, entries 4 and 5).

Next, the effect of temperature was investigated by increasing the reaction temperature from 0 to 60 °C (Table 2, entries 6–8). There was no drastic change in the conversions; therefore, the reactions were conducted at room temperature and 6 mol % for further investigation of the catalytic hydrogenation reaction of 2-acetylpyridine. It was observed that the formation of the silylated byproduct (PhSi(OMe)_3_) increased at elevated temperatures of 40 and 60 °C. The excess production of the silylated byproduct at higher temperatures is proposed to be the consequence of the fast interaction of the agitated solvent molecules with phenylsilane. Visible effervescence was also observed at the beginning of the reaction. The formation of the silylated byproducts containing the −OCH_3_ moieties of the solvent was apparent comparing the m/z values of the byproduct (Figure S4).

The effect of different common organic solvents was also investigated using polar protic and nonpolar solvents, such as water, acetonitrile, ethanol, and methanol, under identical reaction conditions. As evident from the data listed in Table 2 (entry 3 and entries 9–13) the highest conversion of 40% was observed with methanol as the solvent medium followed by other polar protic solvents. This could be due to the effect of size and effective transfer of protons leading to the formation of methoxy-substituted intermediates required for the rapid catalytic process. However, the lower conversion in a solvent like acetonitrile may pertain to the retardation in the effective transfer of hydride to the metal center, which is considered to be the active intermediate for the hydrogenation of ketone in the presence of phenylsilane [24-26]. Keeping the environmental compatibility aspect in concern, we sought to bring about the desired transformations under mild conditions, such as using methanol as the solvent and, avoiding or minimizing the use of other hazardous auxiliaries.

Furthermore, the effect of time on the catalytic hydrogenation was measured starting from 4 h to 72 h (Table 2, entries 14–18). A plot of % alcohol conversion as a function of time is shown in Figure S5. It was observed that, at short time intervals (4–8 h), the reaction could not reach the desired threshold for alcohol generation. Moreover, increasing the reaction time beyond 24 h did not show impressive results that pertain to the persistent interaction of the solvent molecule with phenylsilane-forming methoxy-silylated byproducts before it could act as a reducing agent for the active transfer of its protons to the nickel metal center. We also extended our catalytic analysis to check the efficiency of some common nickel(II) complexes. The nickel–Bipy and nickel–Phen complexes were also examined for their catalytic effectiveness in the hydrogenation of 2-acetylpyridine. Under otherwise identical optimized conditions, the reaction showed only trace amounts of alcohol formation. Additionally, the efficiency of the parent compound Ni(OAc)_2_·4H_2_O was compared to that of catalyst **3** in 2-acetylpyridine reduction, which revealed that Ni(OAc)_2_·4H_2_O did not show any alcohol conversion under analogous reaction conditions. We also conducted a blank experiment in the absence of the catalyst under similar reaction conditions. It was revealed that phenylsilane alone could not reduce the substrate within the stipulated reaction time of 24 h and that catalyst **3** playing a vital role in attaining the required conversion was thus apparent.

**Scheme 2.**
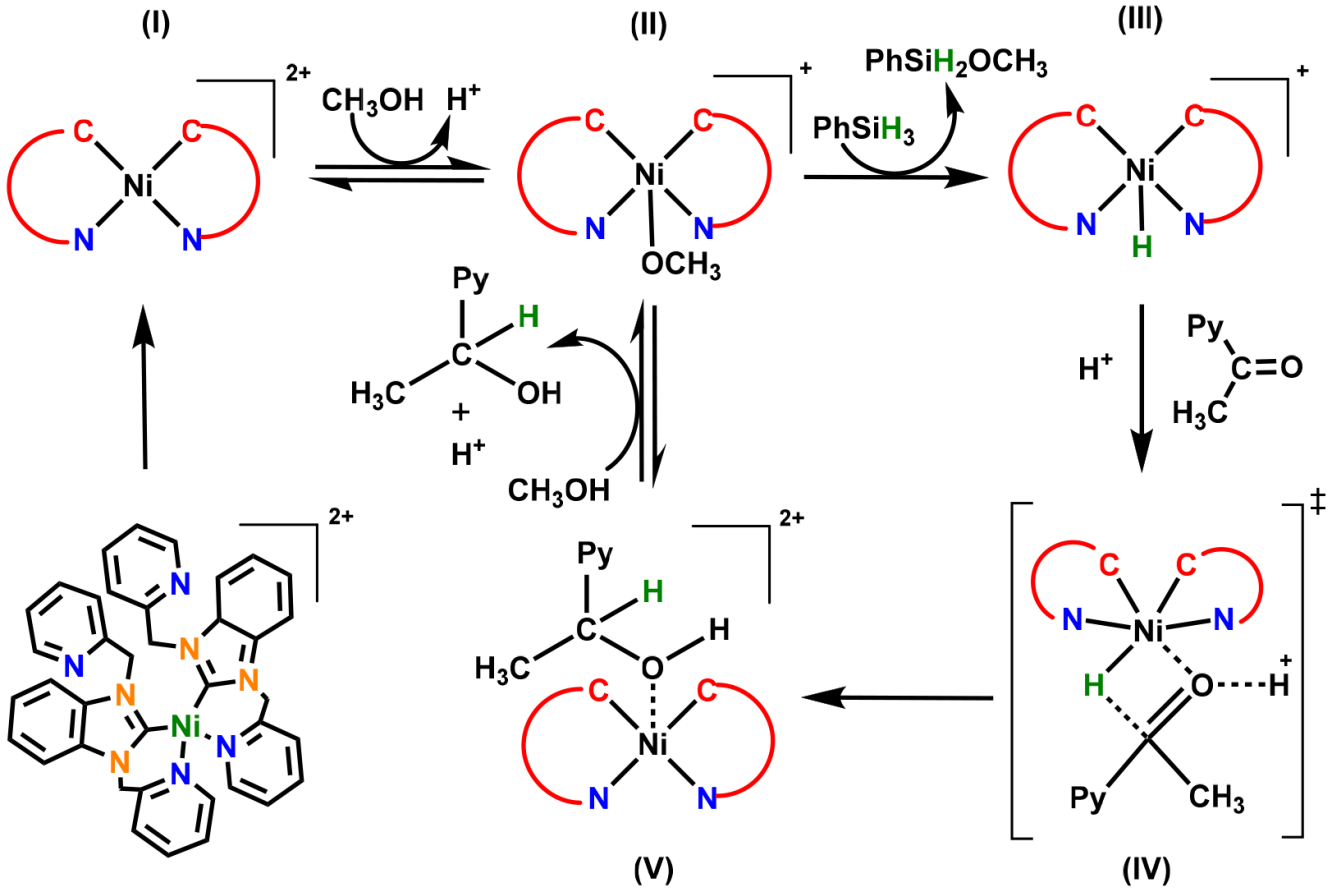
Proposed mechanism for the complex **3** catalyzed hydrogenation of 2-acetylpyridine to 1-(pyridin-2-yl)ethan-1-ol.

Based on our previous experience and several reported methods, we have proposed the mechanism of the reduction of ketones with silane catalyzed by complex **3,** as shown in Scheme 2. In a previous report, Hartwig et al. suggested that there are two possible mechanisms [26]. They suggested that the activation of the carbonyl group takes place by the metal acting as a Lewis acid, and another mechanism proposes that a stepwise pathway could occur involving the formation of a metal hydride. The hydrogenation catalyzed by complex **3** is proposed to follow the formation of a nickel hydride facilitating the formation of the desired alcohol [26,27]. In the first step, complex (I) may abstract a methanol molecule from the solution to form a five-coordinated methoxy-substituted complex (II) and releases one proton in the solution. In the presence of phenylsilane, the −OCH_3_ species gets exchanged with a hydride from phenylsilane, resulting in the formation of nickel hydride species (III). The species (III) interacts with the proton-activated 2-acetylpyridine through a six-membered transition state (IV) to form the hydronickelation intermediate (V). This sterically hindered 5-coordinate species rapidly reacts with solvent, where a proton is abstracted from methanol to form 1-(pyridin-2-yl)ethan-1-ol and regenerate the active five coordinated species (II). This generalized reduction mechanism is consistent with similar pathways reported for other transitionmetal-dependent catalytic systems, where further research into the structure of these intermediates may yield new insight into this process.

The low yields could be attributed to the structure of **3,** where the two NHCs occupy the equatorial plane around the metal. The OCH_3_ in (II) assumed to be in an axial position may attack the silicon atom to facilitate the transfer of the hydride. The metal has no open coordination sites available close to that transfer event; therefore, the only thing around the metal would be CH_3_OH that could be deprotonated by the hydride transfer.

## 3. Experimental Section

### 3.1. Materials

Benzimidazole (ACROS organics), picolyl chloride (TCI), sodium carbonate phenylsilane (Fisher Scientific), ammonium hexafluorophosphate, 2-acetylpyridine (Aldrich), and Nickel(II) acetate tetrahydrate (Alfa Aesar, Haverhill, MA, USA), were used as received. The reagents utilized for this work were of analytical grade and used as received. The solvents used was of HPLC grade and were obtained from Fisher Scientific (Fair Lawn, NJ, USA). The water was deionized initially by using a reverse osmosis system and furiher polished to be ~17 MΩ-cm.

### 3.2. Synthesis of 1,3-Bis(Pyridin-2-Ylmethyl)-1H-Benzo[d]Imidazol-3-Ium Chloride (1) and Synthesis of 1,3-Bis(Pyridin-2-Ylmethyl)-1H-Benzo[d]Imidazol-3-Ium Hexafluorophosphate (2)

Compounds (**1**) and (**2**) were synthesized by following a previously reported method by us [19], as shown in the SI.

### 3.3. Synthesis of Complex (3)

1,3-bis(pyridin-2-ylmethyl)-1H-imidazol-3-ium hexafluorophosphate (0.198 g, 0.5 mmol) was mixed with Ni(OAc)_2_·4H_2_O (0.1244 g, 0.5 mmol) in MeOH (3 mL) and stirred for 1 h at 50 °C. A clear brown solution was obtained and brownish-yellow colored crystals were isolated from this solution in good yields (/yYield: 61%). The crystals were characterized by X-ray diffraction.; λ_max_/nm in CH_3_CN (ε_max_/M^−1^ cm^−1^): 470(273), 303(33,125), 266(38,500), and 202(192,500). ^1^H NMR (DMSO-*d*^6^, 500 MHz): δ = 8.56 (d, J = 7.0 Hz, 1H), 8.28 (d, J = 7.0 Hz, 1H), 8.16 (t, J = (6.0 Hz, 1H), 8.06 (d, J = 7.0 Hz, 1H), 7.99 (d, J = 7.5 Hz, 1H), 7.59 (d, J = 8.0 Hz, 1H), 7.48-7.42 (m, 3H), 7.31 (d, J = 5.5 Hz, 2H), 7.11 (d, J = 7.0 Hz, 1H), 6.90 (d, J = 15.0 Hz, 1H), 6.42 (d, 15.0 Hz, 1H), 5.68 (d, J = 16.0 Hz, 1H), 4.9 (d, J = 16.5 Hz, 1H) ppm (Figure S6). ^13^C NMR (DMSO-*d*_6_, 125 MHz): δ = 172.3, 155.4, 153.9, 153.3, 150.0, 141.8, 138.1, 137.4, 134.6, 133.8, 125.8, 125.6, 124.6, 124.5, 123.8, 122.4, 112.6, 111.7, 49.1 ppm (Figure S7). ESI-MS (m/z): observed 677.21 for [M + H_2_O − 2PF_6_^−^]^2+^; calcd 677.23 for [M + H_2_O − 2PF_6_^−^]^2+^ (Figure S8).

### 3.4. General Procedure for the Hydrogenation of 2-Acetylpyridine

The catalytic reactions were carried out in 10 mL reaction tubes charged with 2-acetylpyridine (0.1 mmol, 0.0112 mL), phenylsilane (0.1 mmol, 0.123 mL), and **3** (0.006 mmol, 6 mol % catalyst loading) in 3 mL H_2_O. The reaction mixture was stirred at room temperature for 24 h under nitrogen atmosphere to yield 1-(pyridin-2-yl)ethan-1-ol as the product. To separate the product, the reaction mixture was extracted in ethyl acetate and the % alcohol conversion was found by GC-MS (Figure S4). The conversions are based on the comparison of the product 1-(pyridin-2-yl)ethan-1-ol to the calibration curve obtained by the known concentration of the pure 1-(pyridin-2-yl)ethan-1-ol.

### 3.5. Experimental Methods

Mass spectrum was obtained employing a Bruker UHPLC microTOF-Q II mass spectrometer system operational in ESI ionization mode. UV-Vis spectra were obtained in an OLIS modernized HP 8452 UV/vis spectrophotometer. IR spectra were recorded in a Thermo Scientific Nicolet 6700 FT-IR. NMR spectra were obtained in a Bruker AVANCE III 500 MHz spectrometer at room temperature. ^1^H chemical shifts are reported with respect to TMS and are referenced to the residual solvent peaks.

The molecular structures of complex **3** weres determined unambiguously by measuring X-ray intensity data at low temperature (T = 100 K), employing a three-circle goniometer platform with a fixed Kappa angle at =54.74 deg Bruker AXS D8 Venture, equipped with a Photon 100 CMOS active pixel sensor detector. Monochromatized copper X-ray radiation (λ = 1.54178 Å) was chosen for the measurement. The structure was solved in a centrosymmetric monoclinic unit cell; Space group: C 2/c, with Z = 8 for the formula unit, C38.50 H33.50 F12 N8 NiO0.50 P2. The crystallographic data are listed in Tables S1 and S2. CCDC number 2245377 contains the supplementary crystallographic data for complex **3.** The GC-MS was recorded using a Shimadzu QP-2010S GC-MS instrument (Kyoto, Japan).

## 4. Conclusions

In conclusion, we have developed a straightforward, efficient, inexpensive, and environmentally clean method for the hydrogenation of 2-acetylpyridine to 1-(pyridin-2-yl)ethan-1-ol using a pendent-type nickel-NHC complex, [Ni(NHC)_2_](PF_6_)_2_, constructed from a benzimidazole moiety used to generate the carbene. In our attempt to study the nickel-NHC catalyzed hydrogenation of 2-acetylpyridine, the reactions were optimized with respect to temperature, time, solvent medium, and catalyst loading giving alcohol conversions up to 40%. The sustainability of the protocol was ensured by conducting the reduction under halogen and additive free environment at ambient room temperature using methanol as the standard solvent. The catalyst is stable, reasonably non-toxic, and of crystalline nature, which offers the advantages of safety and simple handling. The use of environment-friendly and mild reaction conditions are additional considerable features that make the method attractive in terms of environment and economic significance.

## Supplementary Material

Supplementary Information

**Supplementary Materials:** The following supporting information can be downloaded at: https://www.mdpi.com/article/10.3390/inorganics11030120/s1, Table S1: Sample and crystal data for **3**; Table S2: Data collection and structure refinement for **3**; Table S3: Atomic coordinates and equivalent isotropic atomic displacement parameters (Å^2^) for **3**; Table S4: Bond lengths (Å) for **3**; Table S5: Bond angles (°) for **3**; Table S6: Torsion angles (°) for **3**; Table S7: Anisotropic atomic displacement parameters (Å^2^) for **3**; Table S8: Hydrogen atomic coordinates and isotropic atomic displacement parameters (Å^2^) for **3**; Figure S1: Crystal pictures of **3**; Figure S2: Asymmetric unit of **3**; Figure S3: FT-IR spectra of (a) Comparison of compound **2** (red) and complex **3** (black); Figure S4: A typical GC spectrum for the complex **3** catalyzed formation of 1-(pyridin-2-yl)ethan-1-ol from 2-acetylpyridine; Figure S5: Plot for the % conversion of 1-(pyridin-2-yl)ethan-1-ol catalyzed by **3** as a function of time; Figure S6: ^1^H NMR of complex **3**; Figure S7: ^13^C NMR of complex **3**; Figure S8: ESI-MS of complex **3** in CH_3_CN solution.

## Figures and Tables

**Table 1.** Selected bond lengths and (Å) bond angles (°) for **3**.

Bonds	Bond Lengths (Å)
Ni1-C1	1.871(7)
Ni1-C8	1.879(7)
Ni1-N5	1.951(6)
Ni1-N6	1.952(6)
**Bonds**	**Bond Angles (°)**
N5-Ni1-N6	91.7(3)
N6-Ni1-C1	87.8(3)
C1-Ni1-C8	93.8(3)
C8-Ni1-N5	86.7(3)
C8-Ni1-N6	177.9(3)
N5-Ni1-C1	179.1(3)

**Table 2.** Optimization of reaction conditions for complex **3** catalyzed formation of 1-(pyridin-2-yl)ethan-1-ol from 2-acetylpyridine ^a^.

	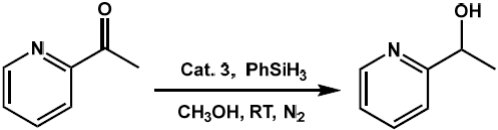				
Entry	Catalyst Loading (Mol %)	Time (h)	Temperature (° C)	Solvent	% Conversion
1	2	24	RT	MeOH	31
2	4	24	RT	MeOH	39
3	6	24	RT	MeOH	40
4	8	24	RT	MeOH	32
5	10	24	RT	MeOH	38
6	6	24	0	MeOH	21
7	6	24	40	MeOH	30
8	6	24	60	MeOH	32
9	6	24	RT	EtOH	9
10	6	24	RT	Propanol	25
11	6	24	RT	Butanol	7
12	6	24	RT	ACN	23
13	6	24	RT	H_2_O	5
14	6	4	RT	MeOH	2
15	6	6	RT	MeOH	5
16	6	8	RT	MeOH	6
17	6	48	RT	MeOH	20
18	6	72	RT	MeOH	28

a Reactions were carried out with 0.1 mmol of the substrate 2-acetylpyridine, 0.1 mmol of phenylsilane, complex 3 as catalyst, and 3 mL of solvent. % conversions reported are averages of 3 or more independent trials with standard deviation of the mean equivalent to less than ±1% error.

## Data Availability

All data are available upon request.
