# Supplementary material for: Nickel(II) N-Heterocyclic Carbene Complex for the Hydrogenation of 2-Acetylpyridine under Mild Conditions
Source: Inorganics (Basel). Author manuscript; Available in PMC 2026 Feb 11. (PMC12889179; doi:10.3390/inorganics11030120)
Supplement: Supplementary Information [file NIHMS2131683-supplement-Supplementary_Information.pdf]

Supplementary Information

Nickel(II) *N*-heterocyclic carbene complex for the hydrogenation of 2-acetylpyridine under mild conditions

Mitu Sharma [1], Amanda M. Perkins [1], Alison K. Duckworth [1], Emily J. Rouse [1], Bruno Donnadieu [1], Bhupendra Adhikari [1], Sean L. Stokes [1,*], and Joseph P. Emerson [1,*]

[1] *Department of Chemistry, Mississippi State University, Mississippi State, MS 39762-9573*

**Correspondence: jemerson@chemistry.msstate.edu; Tel.: 1.662.325.4633*

Table of Contents

Synthesis of **1** and **2**	Page 3
Crystal Structure Report for complex **3**	Page 4
**Figure S1:** Crystal pictures of **3**	Page 5
**Figure S2:** Asymmetric unit of **3**	Page 6
**Table S1:** Sample and crystal data for **3**	Page 7
**Table S2:** Data collection and structure refinement for **3**	Page 8
**Table S3:** Atomic coordinates and equivalent isotropic atomic displacment for **3**	Page 9-11
**Table S4:** Bond lengths for **3**	Page 12-13
**Table S5:** Bond angles for **3**	Page 14-15
**Table S6:** Torsion angles for **3**	Page 16-17
**Table S7:** Anisotropic atomic displacment parameters for **3**	Page 18-20
**Table S8:** Hydrogen atomic coordinates and isotropic atomic displacement for **3**	Page 21-22
**Figure S3:** FT-IR of compound **2** and complex **3**	Page 23
**Figure S4:** A typical GC spectrum for the reduction of 2-acetylpyridine	Page 23
**Figure S5:** Plot for the % conversion of 1-(pyridin-2-yl)ethan-1-ol catalyzed by **3** as a function of time.	Page 24
**Figure S6:** ^{1}H NMR of complex **3**	Page 25
**Figure S7:** ^{13}C NMR of complex **3**	Page 26
**Figure S8:** ESI-MS of complex **3** in CH_3CN solution	Page 27
**References**	Page 27

Synthesis of 1,3-bis(pyridin-2-ylmethyl)-1H-benzo[d]imidazol-3-ium chloride (1)

The compound **1** was synthesized a method previously employed by our group [19]. A mixture of 2-picolyl chloride hydrochloride (5.93 g, 36.15 mmol), benzimidazole (2.13 g, 18.08 mmol), and sodium carbonate (4.55 g, 42.92 mmol) was mixed in 25 mL of ethanol in a 50 mL round bottom flask and refluxed for 36 h [19]. The solvent was removed completely under reduced pressure. The residue was redissolved in di-chloromethane (DCM) (20 mL) and dried over $CaSO_4$. This solution was filtered, and the DCM was removed under reduced pressure. The oily residue obtained was washed with THF (2×10 mL), giving dark brown solid (2).

Synthesis of 1,3-bis(pyridin-2-ylmethyl)-1H-benzo[d]imidazol-3-ium hexafluorophosphate (2)

1,3-Bis(pyridin-2-ylmethyl)-1H-benzo[d]imidazol-3-ium chloride (0.168 g, 0.5 mmol) was dissolved in a minimum amount of H_2O, followed by the addition of 5 equivalents of ammonium hexafluorophosphate (0.407 g, 2.5 mmol). A brown precipitate (2a) was obtained and dried under vacuum. This procedure was previously employed by our group [19].

Crystal Structure Report for complex 3

A yellow single prism like single crystal $C_{38.5}H_{33.5}F_{12}N_8NiO_{0.5}P_2$, approximate dimensions (0.272 x 0.309 x 0.517) mm^3, was selected for the X-ray crystallographic analysis and mounted on a cryoloop using an oil cryoprotectant. The X-ray intensity data was measured at low temperature (T = 270K), using a three circles goniometer Kappa geometry with a fixed Kappa angle at = 54.74 deg Bruker AXS D8 Venture, equipped with a Photon 100 CMOS active pixel sensor detector. A monochromatized Cu X-ray radiation (λ = 0.71073 Å) was selected for the measurement. All frames were integrated with the aid of the Bruker SAINT software [1] using a narrow-frame algorithm. The integration of the data using a monoclinic unit cell yielded a total of 70456 reflections to a maximum θ angle of 26.21° (0.80 Å resolution), of which 8819 were independent (average redundancy 7.989, completeness = 98.4%, R_{int} = 9.68%, R_{sig} = 5.04%) and 6000 (68.03%) were greater than 2σ (F^2). The final cell constants of a = 38.42(4) Å, b = 12.109(11) Å, c = 19.832(18) Å, β = 104.67(3) °, volume = 8926. (15) $Å^3$, are based upon the refinement of the XYZ-centroids of 1357 reflections above 20 σ (I) with 4.383° < 2θ < 48.13°. Data were corrected for absorption effects using the Multi-Scan method (SADABS) [2]. The ratio of minimum to maximum apparent transmission was 0.888. The calculated minimum and maximum transmission coefficients (based on crystal size) are 0.7480 and 0.8550. Structure was solved in a monoclinic unit cell; Space group: C 1 2/c 1, with Z = 8 for the formula unit, C77 H67 F24 N16 Ni2 O P4. Using the Bruker SHELXT Software Package [3], refinement of the structure was carried out by least squares procedures on weighted F^2 values using the SHELXTL-2018/3 [4] included in the APEX4 v2021,10.0, AXS Bruker program [5]. A six members ring was located statistically disordered on two positions and anisotropically refined using 50% occupancy. Two PF_6^- anions were depicted in the unit cell crystallized with the Ni complex, ones was found statistically distributed and anisotropically refined with a ratio of occupancy equal to: 50%. Interatomic lengths and angles were restrained and constraints were added on ADP's parameters. Finally a half molecule of solvent of crystallization; Methanol: CH_3OH was also localized. The final anisotropic full-matrix least-squares refinement on F^2 with 687 variables converged at R1 = 10.71%, for the observed data and wR2 = 30.63% for all data. The goodness-of-fit: GOF was 1.093. The largest peak in the final difference electron density synthesis was 1.442 $e^-/Å^3$ and the largest hole was -1.224 $e^-/Å^3$ with an RMS deviation of 0.116 $e^-/Å^3$. Based on the final model, the calculated density was 1.436 g/cm^3 and F (000), 3924 e^-. Graphics were performed using softwares: Mercury V.4.2.0:

(https://www.ccdc.cam.ac.uk/) and POV-Ray v 3.7: (The Persistence of Vision Raytracer, high quality, Free Software tool).

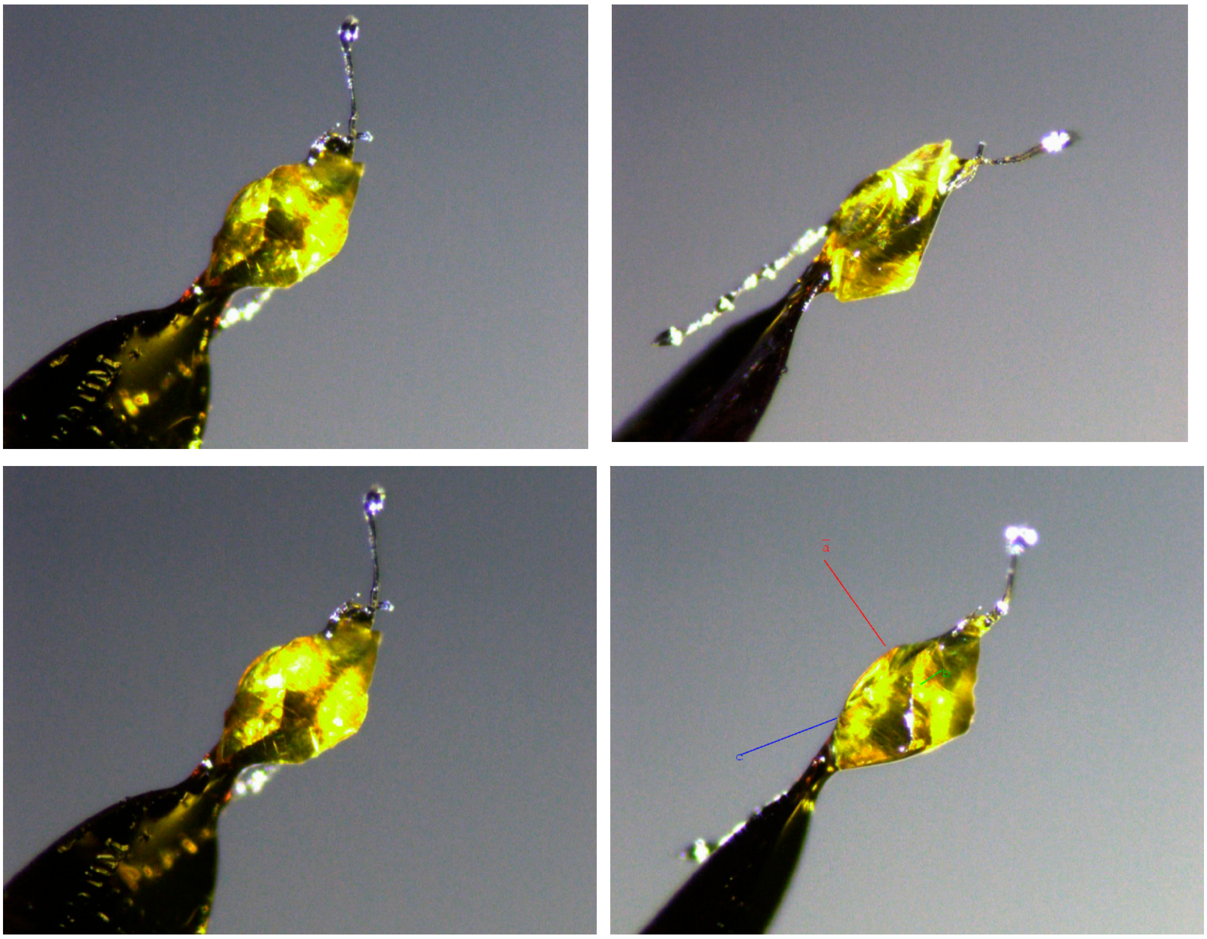

Figure S1. Crystal pictures of **3**

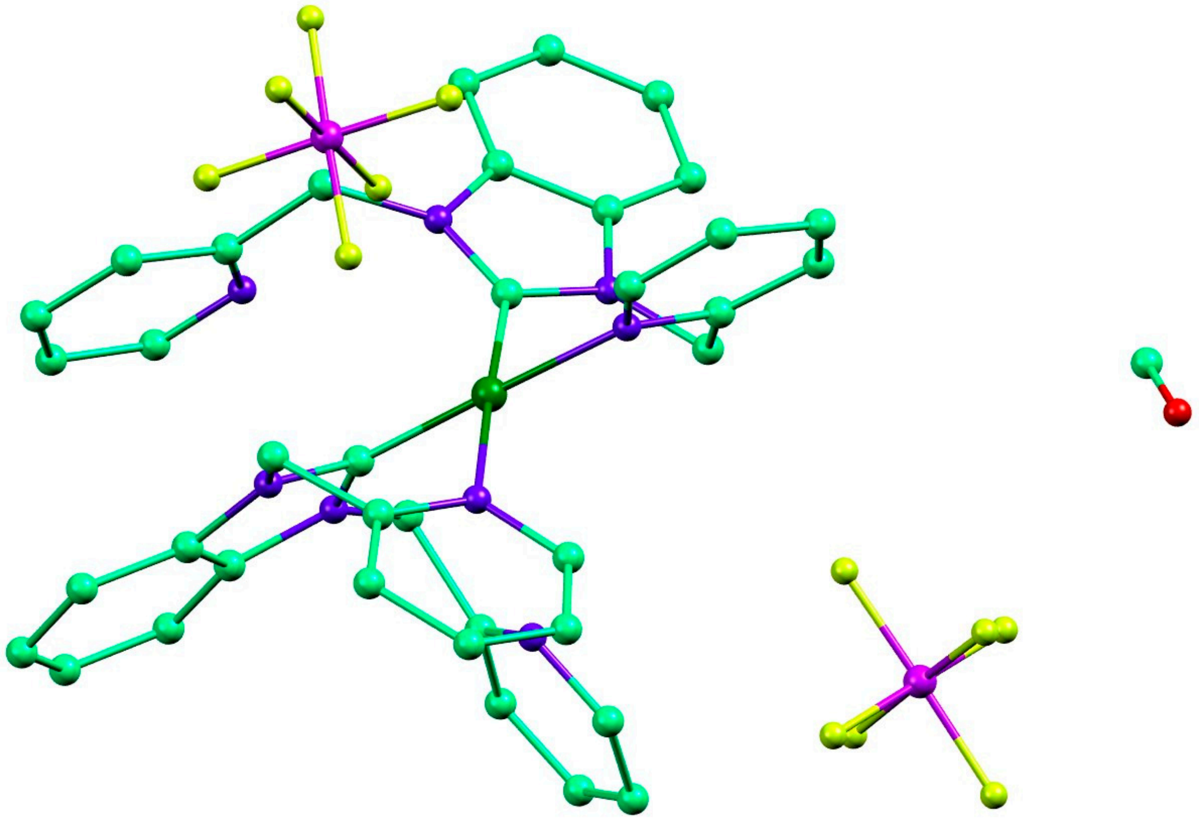

Figure S2. Asymmetric unit of **3**

Table S1. Sample and crystal data for 3.

**Chemical formula**	$C_{38.5}H_{33.5}F_{12}N_8NiO_{0.5}P_2$	
**Formula weight**	964.88 g/mol	
**Temperature**	270(2) K	
**Wavelength**	0.71073 Å	
**Crystal size**	(0.272 x 0.309 x 0.517) mm^3	
**Crystal system**	monoclinic	
**Space group**	C 1 2/c 1	
**Unit cell dimensions**	a = 38.42(4) Å	α = 90°
	b = 12.109(11) Å	β = 104.67(3)°
	c = 19.832(18) Å	γ = 90°
**Volume**	8926.(15) $Å^3$	
**Z**	8	
**Density (calculated)**	1.436 g/cm^3	
**Absorption coefficient**	0.596 mm^{-1}	
**F(000)**	3924	

Table S2. Data collection and structure refinement for 3.

**Theta range for data collection**	1.99 to 26.21°
**Index ranges**	-47<=h<=47, -14<=k<=14, -24<=l<=24
**Reflections collected**	70456
**Independent reflections**	8819 [R(int) = 0.0968]
**Coverage of independent reflections**	98.4%
**Absorption correction**	Multi-Scan
**Max. and min. transmission**	0.8550 and 0.7480
**Refinement method**	Full-matrix least-squares on F^2
**Refinement program**	SHELXL-2019/1 (Sheldrick, 2019)
**Function minimized**	$\Sigma\ w(F_o^2 - F_c^2)^2$
**Data / restraints / parameters**	8819 / 667 / 687
**Goodness-of-fit on F^2**	1.093
**Δ/σ_{max}**	0.001
**Final R indices**	6000 data; $I>2\sigma(I)$ R1 = 0.1071, wR2 = 0.2774
	all data R1 = 0.1500, wR2 = 0.3063
**Weighting scheme**	$w=1/[\sigma^2(F_o^2)+(0.1107P)^2+111.1577P]$
**Extinction coefficient**	0.0017(2)
**Largest diff. peak and hole**	1.442 and -1.224 eÅ^{-3}
**R.M.S. deviation from mean**	0.116 eÅ^{-3}

Table S3. Atomic coordinates and equivalent isotropic atomic displacement parameters (Å^2) for 3.

U(eq) is defined as one third of the trace of the orthogonalized U_{ij} tensor.

	**x/a**	**y/b**	**z/c**	**U(eq)**
Ni1	0.36368(2)	0.36130(7)	0.68595(5)	0.0417(3)
N1	0.40525(16)	0.1522(5)	0.6997(4)	0.0539(16)
N2	0.39552(16)	0.2187(5)	0.7964(4)	0.0515(15)
N3	0.31189(15)	0.1968(5)	0.6057(3)	0.0414(13)
N4	0.33144(17)	0.3217(5)	0.5453(3)	0.0450(14)
N5	0.33906(15)	0.4956(5)	0.6451(3)	0.0423(13)
N6	0.39513(16)	0.4445(5)	0.7617(3)	0.0517(15)
C1	0.38796(18)	0.2335(6)	0.7250(4)	0.0446(16)
C2	0.4179(2)	0.1274(7)	0.8152(5)	0.064(2)
C3	0.4322(3)	0.0786(9)	0.8801(7)	0.091(3)
C4	0.4547(3)	0.9866(10)	0.8804(10)	0.122(5)
C5	0.4612(3)	0.9460(9)	0.8188(9)	0.109(5)
C6	0.4469(3)	0.9942(8)	0.7550(7)	0.085(3)
C7	0.4247(2)	0.0857(7)	0.7538(6)	0.064(2)
C8	0.33354(19)	0.2854(6)	0.6108(4)	0.0428(16)
C9	0.29527(18)	0.1756(6)	0.5342(4)	0.0449(16)
C10	0.2712(2)	0.0946(7)	0.5027(4)	0.057(2)
C11	0.2604(2)	0.1002(8)	0.4287(5)	0.063(2)
C12	0.2731(2)	0.1806(8)	0.3903(4)	0.066(2)
C13	0.2972(2)	0.2611(7)	0.4231(4)	0.057(2)
C14	0.30771(19)	0.2567(6)	0.4956(4)	0.0449(16)
C15	0.3477(2)	0.4285(7)	0.5339(4)	0.060(2)
C16	0.3340(2)	0.5162(6)	0.5763(4)	0.0461(17)
C17	0.3246(2)	0.5652(6)	0.6831(4)	0.0499(17)
C18	0.3056(2)	0.6577(7)	0.6568(5)	0.061(2)
C19	0.3020(3)	0.6834(8)	0.5867(5)	0.076(3)
C20	0.3160(3)	0.6109(7)	0.5455(5)	0.069(2)
C21	0.3851(2)	0.3008(7)	0.8421(4)	0.057(2)
C22	0.4008(2)	0.4125(6)	0.8286(4)	0.060(2)
C23	0.4212(3)	0.4739(9)	0.8835(6)	0.101(4)
C24	0.4383(4)	0.5705(10)	0.8653(8)	0.140(7)
C25	0.4328(3)	0.6005(10)	0.7985(7)	0.111(5)

	x/a	y/b	z/c	U(eq)
C26	0.30239(19)	0.1398(6)	0.6646(4)	0.0453(16)
C27	0.4116(2)	0.5358(7)	0.7472(5)	0.069(2)
C28	0.4076(3)	0.1403(8)	0.6285(6)	0.077(3)
N8A	0.4419(5)	0.3206(18)	0.6491(10)	0.138(8)
C29A	0.4421(5)	0.2166(16)	0.6266(9)	0.082(7)
C30A	0.4665(6)	0.1768(16)	0.5918(11)	0.102(8)
C31A	0.4924(5)	0.249(2)	0.5794(12)	0.127(9)
C32A	0.4926(6)	0.356(2)	0.6023(13)	0.139(9)
C33A	0.4671(6)	0.3896(17)	0.6368(13)	0.148(9)
N8B	0.4272(4)	0.3146(11)	0.6008(8)	0.077(5)
C29B	0.4313(4)	0.2053(12)	0.5966(8)	0.065(5)
C30B	0.4576(5)	0.1564(14)	0.5700(10)	0.093(7)
C31B	0.4811(5)	0.2245(19)	0.5462(10)	0.104(7)
C32B	0.4773(4)	0.3374(18)	0.5501(10)	0.100(7)
C33B	0.4501(5)	0.3796(14)	0.5776(9)	0.105(7)
N7	0.33933(19)	0.9842(5)	0.6474(4)	0.0634(18)
C34	0.32505(19)	0.0401(6)	0.6921(4)	0.0508(18)
C35	0.3294(3)	0.0103(8)	0.7613(4)	0.076(3)
C36	0.3494(3)	0.9158(10)	0.7854(6)	0.105(4)
C37	0.3646(3)	0.8578(8)	0.7400(8)	0.110(5)
C38	0.3590(3)	0.8939(7)	0.6716(7)	0.090(4)
P1	0.28078(6)	0.40409(19)	0.82620(11)	0.0571(6)
F1	0.26481(18)	0.4436(7)	0.8884(3)	0.122(3)
F2	0.2963(2)	0.2993(5)	0.8678(3)	0.117(2)
F3	0.29795(14)	0.3676(4)	0.7639(2)	0.0708(14)
F4	0.26586(17)	0.5120(5)	0.7848(3)	0.102(2)
F5	0.24460(15)	0.3467(6)	0.7908(3)	0.110(2)
F6	0.31741(16)	0.4652(6)	0.8600(4)	0.118(3)
P2A	0.4462(2)	0.7368(6)	0.5841(4)	0.060(2)
F1A	0.4126(3)	0.6714(14)	0.5956(8)	0.148(8)
F2A	0.4298(5)	0.7358(15)	0.5045(5)	0.142(8)
F3A	0.4809(3)	0.7998(15)	0.5728(8)	0.184(10)
F4A	0.4634(5)	0.7356(18)	0.6647(5)	0.191(9)
F5A	0.4279(5)	0.8479(10)	0.5936(12)	0.185(9)
F6A	0.4659(5)	0.6251(11)	0.5761(13)	0.215(11)
P2B	0.4407(3)	0.7199(9)	0.5848(5)	0.102(4)
F1B	0.4244(5)	0.6002(11)	0.5855(11)	0.176(9)
F2B	0.4045(4)	0.7616(17)	0.5383(11)	0.214(11)
F3B	0.4572(6)	0.8417(11)	0.5861(11)	0.199(11)

	x/a	y/b	z/c	U(eq)
F4B	0.4772(4)	0.6783(16)	0.6332(11)	0.213(11)
F5B	0.4551(6)	0.6872(18)	0.5211(9)	0.180(10)
F6B	0.4272(6)	0.7533(17)	0.6507(9)	0.190(9)
O1S	0.3544(3)	0.9991(10)	0.4991(7)	0.068(3)
C1S	0.3407(5)	0.9139(15)	0.4656(9)	0.066(4)

Table S4. Bond lengths (Å) for 3.

Ni1-C1	1.871(7)	Ni1-C8	1.879(7)
Ni1-N5	1.951(6)	Ni1-N6	1.952(6)
N1-C1	1.353(9)	N1-C7	1.398(11)
N1-C28	1.446(12)	N2-C1	1.384(10)
N2-C2	1.394(10)	N2-C21	1.467(10)
N3-C8	1.346(9)	N3-C9	1.423(9)
N3-C26	1.480(9)	N4-C8	1.354(9)
N4-C14	1.403(9)	N4-C15	1.479(9)
N5-C17	1.342(9)	N5-C16	1.353(9)
N6-C27	1.341(10)	N6-C22	1.347(11)
C2-C3	1.396(14)	C2-C7	1.402(13)
C3-C4	1.409(16)	C4-C5	1.40(2)
C5-C6	1.375(18)	C6-C7	1.394(12)
C9-C10	1.385(10)	C9-C14	1.402(10)
C10-C11	1.421(12)	C11-C12	1.399(13)
C12-C13	1.388(12)	C13-C14	1.393(10)
C15-C16	1.528(11)	C16-C20	1.398(11)
C17-C18	1.366(11)	C18-C19	1.397(13)
C19-C20	1.396(14)	C21-C22	1.531(12)
C22-C23	1.387(12)	C23-C24	1.431(18)
C24-C25	1.339(19)	C25-C27	1.375(14)
C26-C34	1.508(10)	C28-C29B	1.463(17)
C28-C29A	1.622(18)	N8A-C29A	1.337(10)
N8A-C33A	1.347(11)	C29A-C30A	1.384(10)
C30A-C31A	1.393(13)	C31A-C32A	1.379(16)
C32A-C33A	1.391(16)	N8B-C29B	1.338(10)
N8B-C33B	1.348(11)	C29B-C30B	1.384(10)
C30B-C31B	1.393(13)	C31B-C32B	1.379(16)
C32B-C33B	1.390(16)	N7-C34	1.338(9)
N7-C38	1.347(10)	C34-C35	1.388(10)
C35-C36	1.393(12)	C36-C37	1.380(15)
C37-C38	1.389(16)	P1-F2	1.548(6)
P1-F5	1.554(6)	P1-F4	1.572(6)
P1-F6	1.581(6)	P1-F1	1.584(5)
P1-F3	1.602(5)	P2A-F2A	1.546(7)
P2A-F5A	1.552(7)	P2A-F4A	1.567(7)

P2A-F6A	1.578(7)	P2A-F1A	1.580(6)
P2A-F3A	1.601(6)	P2B-F2B	1.548(7)
P2B-F5B	1.552(7)	P2B-F4B	1.567(7)
P2B-F6B	1.577(7)	P2B-F1B	1.581(6)
P2B-F3B	1.601(6)	O1S-C1S	1.27(2)

Table S5. Bond angles (°) for 3.

C1-Ni1-C8	93.8(3)	C1-Ni1-N5	179.1(3)
C8-Ni1-N5	86.7(3)	C1-Ni1-N6	87.8(3)
C8-Ni1-N6	177.9(3)	N5-Ni1-N6	91.7(3)
C1-N1-C7	110.7(7)	C1-N1-C28	126.4(7)
C7-N1-C28	122.6(7)	C1-N2-C2	109.4(7)
C1-N2-C21	121.7(6)	C2-N2-C21	128.3(7)
C8-N3-C9	109.4(6)	C8-N3-C26	125.7(6)
C9-N3-C26	124.4(6)	C8-N4-C14	111.2(6)
C8-N4-C15	120.4(6)	C14-N4-C15	127.4(6)
C17-N5-C16	118.5(6)	C17-N5-Ni1	121.2(5)
C16-N5-Ni1	120.2(5)	C27-N6-C22	118.8(7)
C27-N6-Ni1	119.7(6)	C22-N6-Ni1	121.5(5)
N1-C1-N2	106.8(6)	N1-C1-Ni1	133.6(6)
N2-C1-Ni1	118.9(5)	N2-C2-C3	130.9(10)
N2-C2-C7	107.0(8)	C3-C2-C7	122.2(9)
C2-C3-C4	115.8(13)	C5-C4-C3	121.2(13)
C6-C5-C4	122.6(11)	C5-C6-C7	116.8(12)
C6-C7-N1	132.6(10)	C6-C7-C2	121.3(10)
N1-C7-C2	106.1(7)	N3-C8-N4	107.5(6)
N3-C8-Ni1	134.0(5)	N4-C8-Ni1	118.5(5)
C10-C9-C14	122.1(7)	C10-C9-N3	131.1(7)
C14-C9-N3	106.8(6)	C9-C10-C11	114.8(8)
C12-C11-C10	123.0(8)	C13-C12-C11	121.2(8)
C12-C13-C14	116.1(8)	C13-C14-C9	122.8(7)
C13-C14-N4	132.1(7)	C9-C14-N4	105.1(6)
N4-C15-C16	107.8(6)	N5-C16-C20	121.4(7)
N5-C16-C15	116.5(7)	C20-C16-C15	122.1(7)
N5-C17-C18	123.9(8)	C17-C18-C19	118.2(8)
C20-C19-C18	119.1(8)	C19-C20-C16	118.9(8)
N2-C21-C22	108.1(7)	N6-C22-C23	122.5(9)
N6-C22-C21	117.0(6)	C23-C22-C21	120.4(9)
C22-C23-C24	116.4(11)	C25-C24-C23	120.3(10)
C24-C25-C27	119.5(11)	N3-C26-C34	115.0(6)
N6-C27-C25	122.4(10)	N1-C28-C29B	124.7(10)
N1-C28-C29A	102.7(9)	C29A-N8A-C33A	117.3(9)
N8A-C29A-C30A	123.8(9)	N8A-C29A-C28	117.1(13)

C30A-C29A-C28	118.2(14)	C29A-C30A-C31A	118.3(10)
C32A-C31A-	118.7(11)	C31A-C32A-C33A	119.2(10)
N8A-C33A-C32A	122.6(11)	C29B-N8B-C33B	117.3(9)
N8B-C29B-C30B	123.8(9)	N8B-C29B-C28	114.1(12)
C30B-C29B-C28	121.8(12)	C29B-C30B-C31B	118.3(10)
C32B-C31B-	118.7(11)	C31B-C32B-C33B	119.2(10)
N8B-C33B-C32B	122.7(11)	C34-N7-C38	117.5(8)
N7-C34-C35	123.8(7)	N7-C34-C26	117.5(6)
C35-C34-C26	118.7(7)	C34-C35-C36	118.1(9)
C37-C36-C35	118.8(10)	C36-C37-C38	119.3(9)
N7-C38-C37	122.4(10)	F2-P1-F5	93.1(4)
F2-P1-F4	178.7(5)	F5-P1-F4	88.3(4)
F2-P1-F6	88.7(4)	F5-P1-F6	178.0(4)
F4-P1-F6	90.0(4)	F2-P1-F1	90.3(4)
F5-P1-F1	90.5(4)	F4-P1-F1	89.7(3)
F6-P1-F1	90.4(4)	F2-P1-F3	90.4(3)
F5-P1-F3	91.4(3)	F4-P1-F3	89.6(3)
F6-P1-F3	87.7(3)	F1-P1-F3	178.0(4)
F2A-P2A-F5A	92.9(6)	F2A-P2A-F4A	178.7(7)
F5A-P2A-F4A	88.4(6)	F2A-P2A-F6A	88.4(6)
F5A-P2A-F6A	178.3(7)	F4A-P2A-F6A	90.3(6)
F2A-P2A-F1A	90.3(5)	F5A-P2A-F1A	90.2(6)
F4A-P2A-F1A	89.9(5)	F6A-P2A-F1A	90.8(6)
F2A-P2A-F3A	90.0(5)	F5A-P2A-F3A	91.4(6)
F4A-P2A-F3A	89.8(5)	F6A-P2A-F3A	87.5(5)
F1A-P2A-F3A	178.3(6)	F2B-P2B-F5B	92.8(6)
F2B-P2B-F4B	178.9(7)	F5B-P2B-F4B	88.3(6)
F2B-P2B-F6B	88.5(6)	F5B-P2B-F6B	178.5(7)
F4B-P2B-F6B	90.4(6)	F2B-P2B-F1B	90.3(6)
F5B-P2B-F1B	89.8(6)	F4B-P2B-F1B	89.8(6)
F6B-P2B-F1B	90.8(6)	F2B-P2B-F3B	90.0(6)
F5B-P2B-F3B	91.6(6)	F4B-P2B-F3B	89.9(5)
F6B-P2B-F3B	87.8(6)	F1B-P2B-F3B	178.5(7)

Table S6. Torsion angles (°) for 3.

C7-N1-C1-N2	-1.4(8)	C28-N1-C1-N2	-174.2(7)
C7-N1-C1-Ni1	168.9(6)	C28-N1-C1-Ni1	-4.0(12)
C2-N2-C1-N1	0.4(8)	C21-N2-C1-N1	171.7(6)
C2-N2-C1-Ni1	-171.6(5)	C21-N2-C1-Ni1	-0.3(9)
C8-Ni1-C1-N1	53.2(7)	N6-Ni1-C1-N1	-125.5(7)
C8-Ni1-C1-N2	-137.5(6)	N6-Ni1-C1-N2	43.8(6)
C1-N2-C2-C3	-178.6(9)	C21-N2-C2-C3	10.8(14)
C1-N2-C2-C7	0.8(8)	C21-N2-C2-C7	-169.8(7)
N2-C2-C3-C4	-179.1(9)	C7-C2-C3-C4	1.6(15)
C2-C3-C4-C5	-1.7(18)	C3-C4-C5-C6	2.(2)
C4-C5-C6-C7	-1.1(18)	C5-C6-C7-N1	-178.0(9)
C5-C6-C7-C2	1.1(14)	C1-N1-C7-C6	-179.0(9)
C28-N1-C7-C6	-5.8(14)	C1-N1-C7-C2	1.8(8)
C28-N1-C7-C2	175.0(8)	N2-C2-C7-C6	179.2(7)
C3-C2-C7-C6	-1.4(13)	N2-C2-C7-N1	-1.5(8)
C3-C2-C7-N1	177.9(8)	C9-N3-C8-N4	-0.1(7)
C26-N3-C8-N4	-171.7(6)	C9-N3-C8-Ni1	-179.9(5)
C26-N3-C8-Ni1	8.5(11)	C14-N4-C8-N3	0.5(8)
C15-N4-C8-N3	170.3(6)	C14-N4-C8-Ni1	-179.6(5)
C15-N4-C8-Ni1	-9.8(9)	C1-Ni1-C8-N3	52.1(7)
N5-Ni1-C8-N3	-128.6(7)	C1-Ni1-C8-N4	-127.7(6)
N5-Ni1-C8-N4	51.6(5)	C8-N3-C9-C10	179.9(8)
C26-N3-C9-C10	-8.4(12)	C8-N3-C9-C14	-0.4(8)
C26-N3-C9-C14	171.3(6)	C14-C9-C10-C11	0.3(11)
N3-C9-C10-C11	179.9(7)	C9-C10-C11-C12	0.3(12)
C10-C11-C12-C13	-0.3(14)	C11-C12-C13-C14	-0.2(12)
C12-C13-C14-C9	0.8(11)	C12-C13-C14-N4	179.1(8)
C10-C9-C14-C13	-0.9(11)	N3-C9-C14-C13	179.4(7)
C10-C9-C14-N4	-179.6(7)	N3-C9-C14-N4	0.7(7)
C8-N4-C14-C13	-179.3(8)	C15-N4-C14-C13	11.8(13)
C8-N4-C14-C9	-0.8(8)	C15-N4-C14-C9	-169.7(7)
C8-N4-C15-C16	-51.4(9)	C14-N4-C15-C16	116.6(8)
C17-N5-C16-C20	3.5(10)	Ni1-N5-C16-C20	179.0(6)
C17-N5-C16-C15	-174.0(6)	Ni1-N5-C16-C15	1.4(8)
N4-C15-C16-N5	54.5(9)	N4-C15-C16-C20	-123.0(8)
C16-N5-C17-C18	-1.5(11)	Ni1-N5-C17-C18	-176.9(6)

N5-C17-C18-C19	-2.1(12)	C17-C18-C19-C20	3.5(14)
C18-C19-C20-C16	-1.6(14)	N5-C16-C20-C19	-2.0(13)
C15-C16-C20-C19	175.4(8)	C1-N2-C21-C22	-54.8(9)
C2-N2-C21-C22	114.7(8)	C27-N6-C22-C23	4.0(13)
Ni1-N6-C22-C23	-176.0(8)	C27-N6-C22-C21	-172.9(7)
Ni1-N6-C22-C21	7.1(10)	N2-C21-C22-N6	49.2(9)
N2-C21-C22-C23	-127.8(10)	N6-C22-C23-C24	-4.1(17)
C21-C22-C23-C24	172.7(11)	C22-C23-C24-C25	3.(2)
C23-C24-C25-C27	-2.(2)	C8-N3-C26-C34	-95.9(8)
C9-N3-C26-C34	93.7(8)	C22-N6-C27-C25	-2.8(14)
Ni1-N6-C27-C25	177.2(9)	C24-C25-C27-N6	2.(2)
C1-N1-C28-C29B	76.7(14)	C7-N1-C28-C29B	-95.4(13)
C1-N1-C28-C29A	85.8(11)	C7-N1-C28-C29A	-86.2(11)
C33A-N8A-C29A-C30A	-0.01(16)	C33A-N8A-C29A-C28	-169.0(15)
N1-C28-C29A-N8A	-52.6(13)	N1-C28-C29A-C30A	137.7(10)
N8A-C29A-C30A-C31A	-0.01(16)	C28-C29A-C30A-C31A	168.9(15)
C29A-C30A-C31A-C32A	0.0(4)	C30A-C31A-C32A-C33A	0.0(5)
C29A-N8A-C33A-C32A	0.0(4)	C31A-C32A-C33A-N8A	0.0(5)
C33B-N8B-C29B-C30B	0.02(16)	C33B-N8B-C29B-C28	174.7(13)
N1-C28-C29B-N8B	-55.0(15)	N1-C28-C29B-C30B	119.8(10)
N8B-C29B-C30B-C31B	-0.02(16)	C28-C29B-C30B-C31B	-174.3(14)
C29B-C30B-C31B-C32B	0.1(3)	C30B-C31B-C32B-C33B	-0.1(5)
C29B-N8B-C33B-C32B	-0.1(4)	C31B-C32B-C33B-N8B	0.1(5)
C38-N7-C34-C35	0.1(12)	C38-N7-C34-C26	-178.8(7)
N3-C26-C34-N7	-28.3(9)	N3-C26-C34-C35	152.7(7)
N7-C34-C35-C36	-1.0(13)	C26-C34-C35-C36	177.9(8)
C34-C35-C36-C37	1.4(15)	C35-C36-C37-C38	-0.9(17)
C34-N7-C38-C37	0.4(14)	C36-C37-C38-N7	0.1(17)

Table S7. Anisotropic atomic displacement parameters (Å^2) for 3.

The anisotropic atomic displacement factor exponent takes the form: $-2\pi^2[h^2a^{*2} U_{11} + ... + 2 h k a^*b^*U_{12}]$

	U_{11}	U_{22}	U_{33}	U_{23}	U_{13}	U_{12}
Ni1	0.0434(5)	0.0376(5)	0.0429(5)	-0.0012(4)	0.0091(4)	-0.0031(4)
N1	0.046(3)	0.043(3)	0.076(5)	-0.002(3)	0.020(3)	0.004(3)
N2	0.045(3)	0.038(3)	0.067(4)	-0.001(3)	0.007(3)	0.002(3)
N3	0.044(3)	0.037(3)	0.043(3)	-0.005(2)	0.011(3)	-0.001(2)
N4	0.061(4)	0.042(3)	0.036(3)	-0.004(3)	0.019(3)	-0.004(3)
N5	0.045(3)	0.039(3)	0.041(3)	-0.003(2)	0.007(2)	-0.008(2)
N6	0.046(3)	0.046(4)	0.057(4)	-0.002(3)	0.003(3)	-0.001(3)
C1	0.040(4)	0.044(4)	0.049(4)	-0.002(3)	0.011(3)	-0.005(3)
C2	0.045(4)	0.042(4)	0.090(7)	0.013(4)	-0.010(4)	0.001(3)
C3	0.083(7)	0.070(7)	0.109(9)	0.005(6)	0.002(6)	0.015(6)
C4	0.092(9)	0.062(7)	0.180(15)	0.030(9)	-0.026(9)	0.024(6)
C5	0.082(8)	0.056(7)	0.172(14)	-0.007(8)	0.002(9)	0.025(6)
C6	0.061(6)	0.051(5)	0.138(10)	0.003(6)	0.014(6)	0.014(4)
C7	0.041(4)	0.045(4)	0.105(7)	-0.006(5)	0.018(4)	0.000(3)
C8	0.045(4)	0.035(4)	0.050(4)	-0.001(3)	0.015(3)	0.003(3)
C9	0.041(4)	0.048(4)	0.046(4)	-0.009(3)	0.011(3)	0.004(3)
C10	0.054(4)	0.063(5)	0.055(5)	-0.020(4)	0.016(4)	-0.012(4)
C11	0.055(5)	0.073(6)	0.057(5)	-0.023(4)	0.006(4)	-0.008(4)
C12	0.067(5)	0.078(6)	0.044(4)	-0.019(4)	-0.004(4)	0.011(5)
C13	0.065(5)	0.062(5)	0.042(4)	-0.006(4)	0.011(4)	0.010(4)
C14	0.051(4)	0.043(4)	0.037(4)	-0.003(3)	0.005(3)	0.003(3)
C15	0.071(5)	0.055(5)	0.059(5)	0.007(4)	0.026(4)	-0.011(4)
C16	0.057(4)	0.043(4)	0.038(4)	-0.001(3)	0.010(3)	-0.011(3)
C17	0.051(4)	0.046(4)	0.053(4)	-0.006(3)	0.013(3)	-0.003(3)
C18	0.059(5)	0.050(5)	0.072(6)	-0.010(4)	0.012(4)	0.002(4)
C19	0.091(7)	0.052(5)	0.078(7)	0.006(5)	0.005(5)	0.009(5)
C20	0.097(7)	0.047(5)	0.056(5)	0.015(4)	0.006(5)	-0.008(4)
C21	0.065(5)	0.054(5)	0.046(4)	-0.002(4)	0.006(4)	0.012(4)
C22	0.060(5)	0.043(4)	0.061(5)	-0.009(4)	-0.014(4)	0.003(4)
C23	0.121(9)	0.073(7)	0.072(7)	-0.002(5)	-0.041(6)	-0.010(6)
C24	0.153(13)	0.073(8)	0.134(12)	-0.002(8)	-0.072(10)	-0.028(8)
C25	0.106(9)	0.072(7)	0.120(10)	0.022(7)	-0.038(8)	-0.032(6)

	U_{11}	U_{22}	U_{33}	U_{23}	U_{13}	U_{12}
C26	0.050(4)	0.042(4)	0.047(4)	-0.003(3)	0.017(3)	-0.007(3)
C27	0.059(5)	0.048(5)	0.091(7)	0.001(5)	0.002(5)	-0.015(4)
C28	0.093(7)	0.065(6)	0.093(7)	0.001(5)	0.059(6)	0.017(5)
N8A	0.147(17)	0.124(15)	0.159(18)	-0.006(16)	0.068(15)	-0.032(14)
C29A	0.085(14)	0.076(12)	0.108(16)	0.013(12)	0.064(13)	0.003(11)
C30A	0.093(15)	0.118(16)	0.116(17)	0.009(14)	0.063(14)	0.011(13)
C31A	0.090(15)	0.167(19)	0.15(2)	0.016(17)	0.071(15)	-0.002(15)
C32A	0.102(16)	0.154(18)	0.17(2)	0.025(18)	0.054(15)	-0.036(15)
C33A	0.149(19)	0.132(17)	0.17(2)	0.003(17)	0.058(17)	-0.041(16)
N8B	0.077(10)	0.054(8)	0.109(13)	0.013(9)	0.040(9)	-0.006(8)
C29B	0.072(12)	0.055(10)	0.068(12)	-0.013(9)	0.019(10)	0.004(9)
C30B	0.082(14)	0.117(15)	0.090(15)	-0.023(12)	0.041(12)	-0.011(12)
C31B	0.076(13)	0.138(17)	0.103(16)	0.000(15)	0.030(12)	-0.019(13)
C32B	0.072(12)	0.132(16)	0.098(15)	0.017(14)	0.024(11)	-0.038(12)
C33B	0.092(14)	0.103(14)	0.123(17)	0.032(13)	0.032(13)	-0.017(12)
N7	0.067(4)	0.042(4)	0.080(5)	-0.012(3)	0.018(4)	-0.002(3)
C34	0.044(4)	0.046(4)	0.058(5)	0.005(4)	0.006(3)	-0.010(3)
C35	0.076(6)	0.076(6)	0.066(6)	0.024(5)	0.001(5)	-0.015(5)
C36	0.094(8)	0.095(9)	0.101(9)	0.047(8)	-0.023(7)	-0.019(7)
C37	0.072(7)	0.049(6)	0.183(15)	0.023(8)	-0.017(8)	0.003(5)
C38	0.079(7)	0.046(5)	0.133(11)	0.001(6)	0.005(7)	0.006(5)
P1	0.0550(12)	0.0713(14)	0.0461(11)	-0.0077(10)	0.0149(9)	0.0098(10)
F1	0.126(5)	0.190(8)	0.058(3)	0.002(4)	0.042(3)	0.076(5)
F2	0.155(6)	0.110(5)	0.091(4)	0.037(4)	0.043(4)	0.055(5)
F3	0.086(3)	0.077(3)	0.058(3)	-0.020(2)	0.033(3)	0.001(3)
F4	0.111(5)	0.100(5)	0.107(5)	0.033(4)	0.047(4)	0.043(4)
F5	0.075(4)	0.156(6)	0.093(4)	0.004(4)	0.011(3)	-0.041(4)
F6	0.086(4)	0.134(6)	0.120(5)	-0.067(5)	0.000(4)	-0.011(4)
P2A	0.066(4)	0.063(4)	0.051(4)	-0.014(3)	0.012(3)	-0.004(3)
F1A	0.088(10)	0.142(16)	0.21(2)	0.084(16)	0.027(11)	-0.036(11)
F2A	0.164(18)	0.187(18)	0.047(7)	-0.008(9)	-0.022(10)	-0.045(15)
F3A	0.135(15)	0.26(2)	0.168(16)	-0.104(16)	0.059(13)	-0.111(16)
F4A	0.19(2)	0.28(3)	0.091(11)	0.005(14)	0.020(13)	0.007(19)
F5A	0.22(2)	0.092(12)	0.24(2)	-0.020(14)	0.05(2)	0.045(14)
F6A	0.20(2)	0.161(18)	0.26(3)	-0.009(19)	0.03(2)	0.091(16)
P2B	0.096(7)	0.125(9)	0.093(7)	-0.025(6)	0.037(5)	-0.022(6)
F1B	0.19(2)	0.131(16)	0.22(2)	-0.021(16)	0.077(17)	-0.045(15)
F2B	0.135(16)	0.25(2)	0.22(2)	0.01(2)	-0.032(16)	0.026(17)
F3B	0.17(2)	0.110(14)	0.29(3)	-0.011(16)	0.00(2)	-0.032(15)

	U_{11}	U_{22}	U_{33}	U_{23}	U_{13}	U_{12}
F4B	0.146(16)	0.22(2)	0.23(2)	-0.076(19)	-0.020(17)	0.095(16)
F5B	0.174(19)	0.28(3)	0.115(15)	-0.070(16)	0.098(14)	-0.071(19)
F6B	0.25(2)	0.20(2)	0.144(16)	-0.040(15)	0.080(16)	0.083(18)
O1S	0.075(7)	0.061(6)	0.073(7)	0.009(5)	0.026(6)	0.005(5)
C1S	0.079(9)	0.062(8)	0.061(8)	0.005(6)	0.023(7)	0.015(7)

Table S8. Hydrogen atomic coordinates and isotropic atomic displacement parameters (Å^2) for 3.

	x/a	y/b	z/c	U(eq)
H3	0.4272	0.1053	0.9206	0.109000
H4	0.4655	-0.0478	0.9225	0.146000
H5	0.4757	-0.1159	0.8210	0.131000
H6	0.4518	-0.0330	0.7145	0.102000
H10	0.2627	0.0407	0.5279	0.068000
H11	0.2441	0.0480	0.4048	0.076000
H12	0.2652	0.1802	0.3419	0.079000
H13	0.3059	0.3148	0.3980	0.068000
H15A	0.3409	0.4475	0.4848	0.072000
H15B	0.3737	0.4238	0.5488	0.072000
H17	0.3277	0.5496	0.7302	0.060000
H18	0.2954	0.7024	0.6848	0.073000
H19	0.2904	0.7479	0.5677	0.092000
H20	0.3133	0.6253	0.4984	0.082000
H21A	0.3944	0.2797	0.8905	0.068000
H21B	0.3591	0.3055	0.8322	0.068000
H23	0.4237	0.4534	0.9297	0.121000
H24	0.4532	0.6126	0.9001	0.168000
H25	0.4433	0.6645	0.7868	0.134000
H26A	0.2774	0.1170	0.6499	0.054000
H26B	0.3045	0.1922	0.7025	0.054000
H27	0.4086	0.5562	0.7009	0.083000
H28A	0.3860	0.1669	0.5959	0.092000
H28B	0.4117	0.0639	0.6178	0.092000
H30A	0.4656	0.1036	0.5771	0.123000
H31A	0.5092	0.2250	0.5562	0.153000
H32A	0.5097	0.4063	0.5946	0.167000
H33A	0.4674	0.4624	0.6520	0.177000
H30B	0.4594	0.0800	0.5681	0.111000
H31B	0.4991	0.1945	0.5279	0.125000
H32B	0.4927	0.3847	0.5347	0.120000
H33B	0.4477	0.4558	0.5800	0.126000
H35	0.3193	0.0521	0.7908	0.091000
H36	0.3526	-0.1077	0.8312	0.126000

	x/a	y/b	z/c	U(eq)
H37	0.3783	-0.2049	0.7551	0.132000
H38	0.3693	-0.1458	0.6414	0.108000
H1S1	0.3553	0.8907	0.4355	0.099000
H1S2	0.3169	0.9308	0.4383	0.099000
H1S3	0.3395	0.8557	0.4978	0.099000

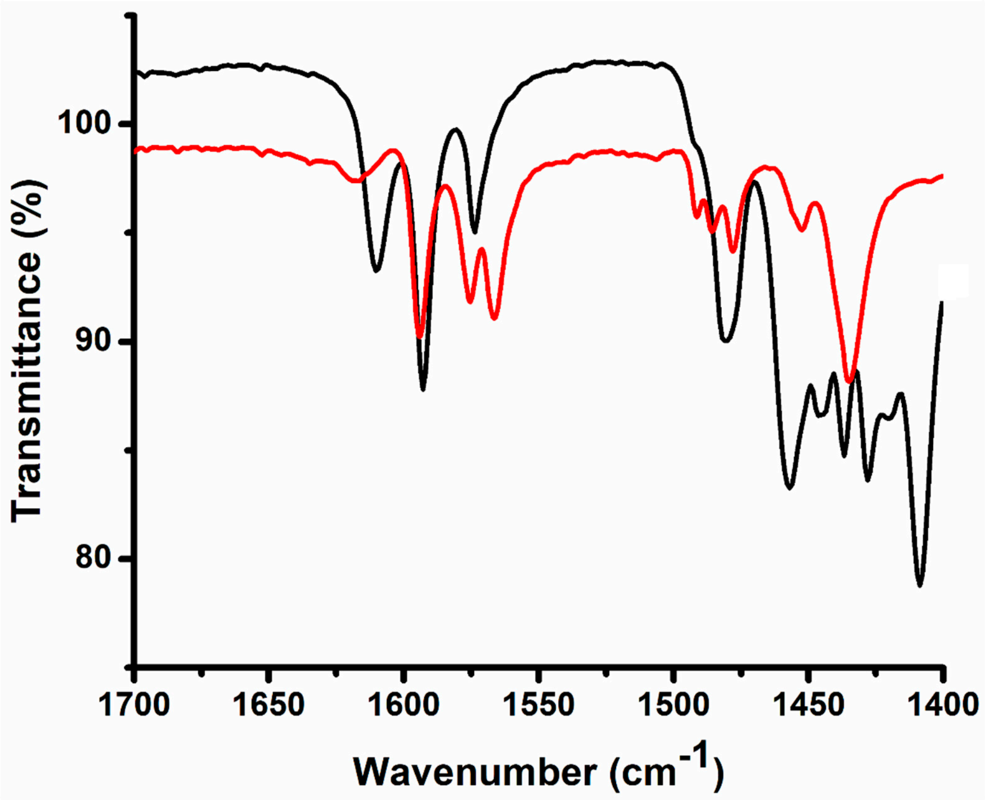

Figure S3. FT-IR spectra of (a) Comparison of compound **2** (red) and complex **3** (black).

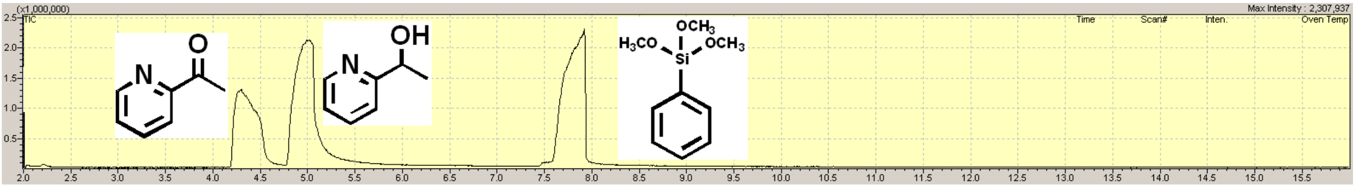

Figure S4. A typical GC spectrum for the complex **3** catalyzed formation of 1-(pyridin-2-yl)ethan-1-ol from 2-acetylpyridine.

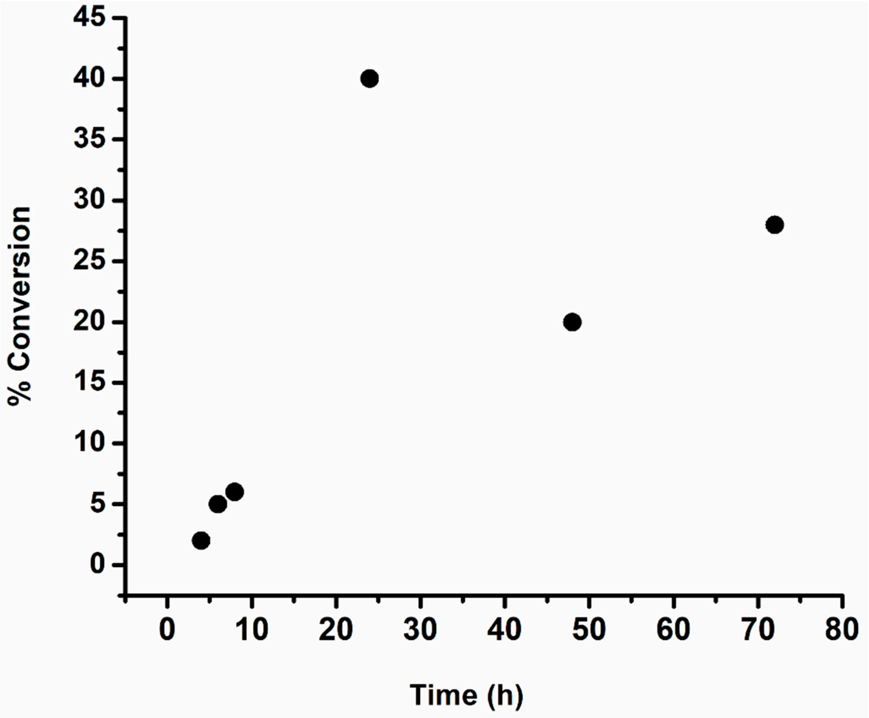

Figure S5. Plot for the % conversion of 1-(pyridin-2-yl)ethan-1-ol catalyzed by **3** as a function of time.

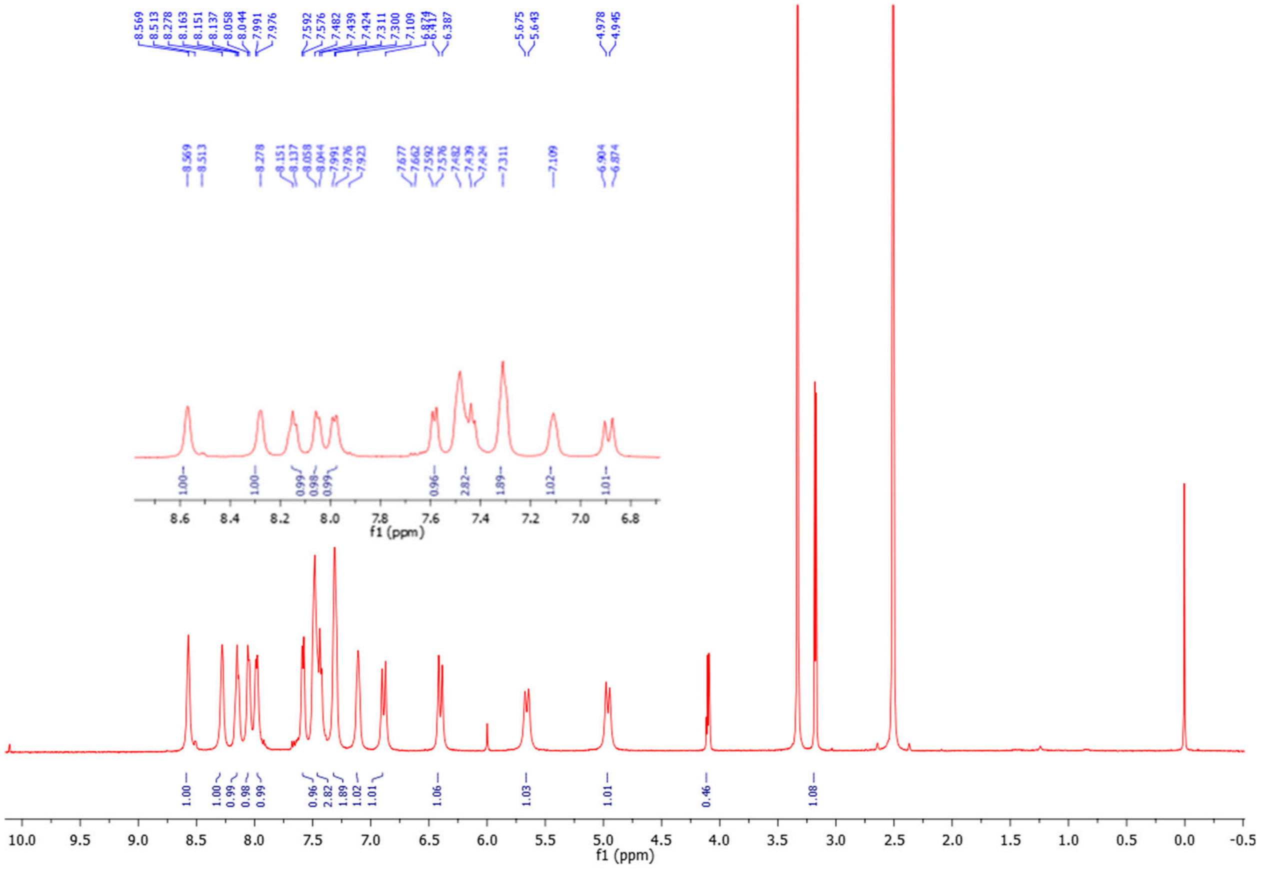

Figure S6. ^{1}H NMR of complex **3**.

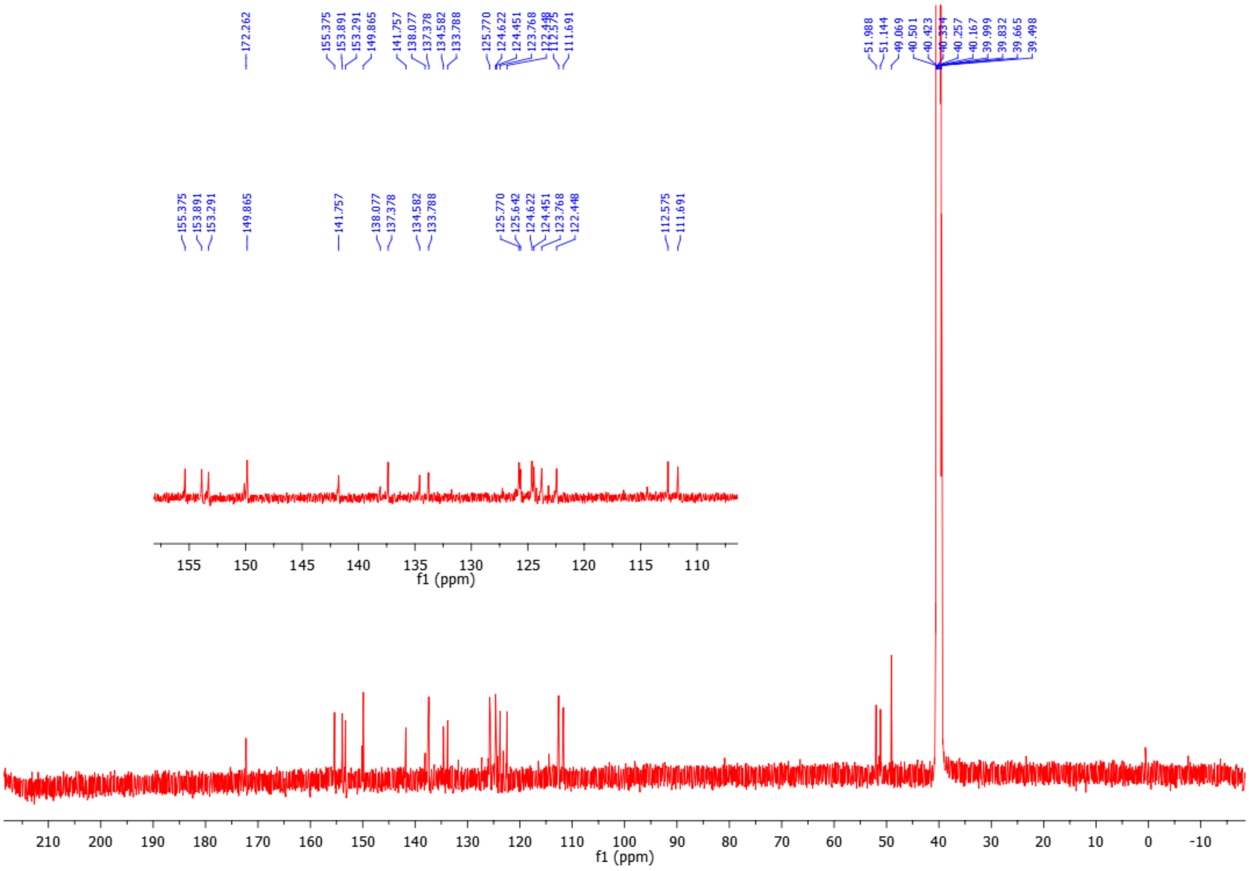

Figure S7. ^{13}C NMR of complex **3**.

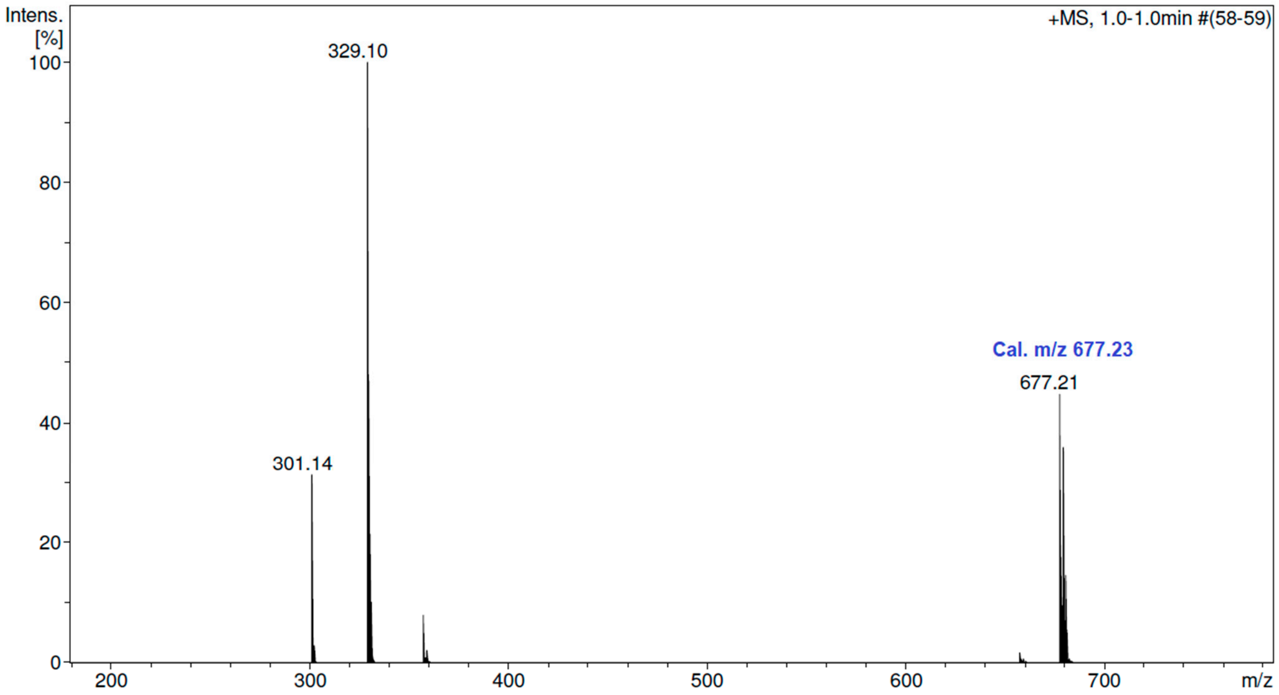

Figure S8. ESI-MS of complex **3** in CH_3CN solution.

References

R1 Saint Program included in the package software: APEX4 v2021.10.0.

R2 Bruker, *Program name.*Bruker AXS Inc. **2001**, Madison, Wisconsin, USA.

R3 SHELXT-Integrated space-group and crystal-structure determination Sheldrick, G. M. Acta Crystallogr., Sect. A **2015**, *A71*, 3-8.

R4, Ver, G. M. SHELXTL Sheldrick , Acta Crystallographica. Sect C Structural Chemistry, **2018**, *71*, 3 - 8.

R5 APEX4 v2021, 10.0, AXS Bruker program.
